# Medium-term results after implant of a new generation stentless aortic prosthesis: hemodynamic performance of medtronic 3F® stentless equine pericardial aortic valve

**DOI:** 10.1186/1749-8090-8-S1-O320

**Published:** 2013-09-11

**Authors:** G Stefanelli, D Gabbieri, C Labia, G D'Anniballe, D Sarandria, R Santangelo, G Gioia

**Affiliations:** 1 Cardiac Surgery, Hesperia Hospital, Modena, Italy

## Background

Aim of this study was to evaluate the clinical and hemodynamic results at 5 years of Medtronic 3F® stentless equine pericardial aortic valve.

## Methods

Between March 2007 and December 2012 a total of 120 consecutive patients affected by aortic valve disease received a 3F valve at our unit. The size ranged between 21 and 29, with prevalence of 23 and 25 implants .Mean age at operation was 75,6±8,0 years, 54% were males, the mean logistic EuroScore was 8,7±5,2 and 47% received concomitant procedures. For isolated replacements the mean ECC time was 90’±10’, Cross Clamp time 72’±8’.In 12 pts the aortic prosthesis was included in a Dacron tube straight graft for a Bentall operation.

## Results

Early mortality in isolated AVR was 2,1% and 3,1% in the entire group. There have been 6 late all-causes deaths (5,6%) ,with a survival of 90% at 5 yrs. 91% of patients are in NYHA class I or II. Actuarial freedom from reoperation for structural deterioration is 100% at 5 years. Freedom from endocarditis and thromboembolic events was 96% and 99% respectively. The 72 patients evaluated under exercise at 75w protocol showed a moderate increase in the MPG (from 7,8±3,3mmHg to 11,6±4.0mmHg) for the entire series at five years follow up.

## Conclusions

The 3F valve shows excellent hemodynamics, durability and freedom from structural deterioration at 5 yrs follow-up. Freedom from endocarditis and thromboembolic events looks satisfactory. Longer follow-up times are needed for a better evaluation of this interesting new generation of user friendly aortic valve substitute.

